# Microorganisms: Good or Evil, MIRRI Provides Biosecurity Awareness

**DOI:** 10.1007/s00284-016-1181-y

**Published:** 2016-12-26

**Authors:** David Smith, Dunja Martin, Tatyana Novossiolova

**Affiliations:** 1 CABI, Bakeham Lane, Egham, Surrey TW20 9TY UK; 2 Leibniz-Institut DSMZ-Deutsche Sammlung von Mikro-organismen und Zellkulturen GmbH, Inhoffenstraße 7 B, 38124 Brunswick, Germany; 3 University of Bradford, Bradford, West Yorkshire BD7 1DP UK

**Keywords:** Biorisk, Biosecurity, Biological resource centre, Code of conduct, Microbial resources research infrastructure, Microorganisms

## Abstract

The life-science community is a key stakeholder in the effort to ensure that the advances in biotechnology are not misused. Unfortunately, to date, the engagement of life scientists with issues of biosecurity has been limited. Microorganisms have been harnessed for the benefit of humankind but in the wrong hands could be used in direct or indirect acts against humans, livestock, crops, food, water infrastructure and other economically valuable entities. The Microbial Resources Research Infrastructure in its preparatory phase has addressed the topic implementing a code of conduct as part of its programme of prevention of malicious use and continues to work with the international community to raise awareness of best practice to avoid misuse of microorganisms. Biosecurity has become a major concern for several countries creating numerous activities to put in place counter measures, risk assessment, legislation and emergency response. The goal is to implement measures to protect us against malicious use of microorganisms, their products, information and technology transfer. Through this paper, we wish to discuss some of the activities that are underway, mention key educational tools and provide scientists with information on addressing biosecurity issues.

## Introduction

Microorganisms are a vital component of the world’s biodiversity. They are involved in nutrient recycling (e.g. breaking down complex plant and animal remains), beneficial mutualistic relationships (e.g. nitrogen fixation, animal digestion, mycorrhiza) and production of atmospheric oxygen. Moreover, they are pathogens of pests and disease-causing organisms and, hence, may be harnessed by man for the biological control of pests in integrated pest management programmes. Their other uses include production of natural products (e.g. valuable drugs, enzymes and metabolites) for pharmaceutical, food and other applications, composting, bioremediation and detoxification of wastes. They play a major role in soil fertility and plant and animal health and are employed in diagnostics, efficacy testing of drugs, biocides, vaccine production and disinfectants or as reference strains. Harnessed correctly they can provide solutions to the sustainable development goals, for example making contributions to alleviation of poverty and hunger, sources of energy and in the improvement of health.

Worldwide, there are thousands of organisations of varying sizes that handle microorganisms. These organisations range from medical centres, universities and research institutes, veterinary diagnostic laboratories, phytopathology facilities, research and development facilities of in vitro diagnostic, vaccine manufacturers and their production plants to comprehensive biological resource centres. All of these organisations are requested to manage the associated biorisks, which includes mitigating the risk of an intentional misuse or unintentional release of a microorganism from a facility that may result in deleterious impact to human health, the environment and/or the economy. Additionally, the public face of microbiology is tainted by the ability of some to cause deterioration, death and destruction. Forgotten is Fleming’s penicillin producer or the immunosuppressant cyclosporin and replaced by the thought of potential misuse. Are they our friends or our foes? Certainly the results of microbiological research tell us that we are making tremendous advances in knowledge and our ability to harness the potential of microorganisms. Microbiologists need to be aware of the needs of biosecurity in order to ensure our security from misuse while continuing the discovery path towards the next antimicrobial against the ever increasing resistant disease-causing organisms.

Many countries, including many developing economies, lack national regulations that establish requirements for managing biorisks. Biosecurity has become a major concern for several countries creating numerous activities to put in place counter measures, risk assessment, legislation and emergency response. The Organisation for Economic Co-operation and Development (OECD) Best Practice Guidelines offers a definition for ‘biosecurity’ in the context of a Biological Resource Centres (BRCs) which states that it is institutional and personal security measures and procedures designed to prevent the loss, theft, misuse, diversion or intentional release of pathogens, or parts of them, and toxin-producing organisms, as well as such toxins that are held, transferred and/or supplied by BRCs [15]. In this guidance document, BRCs distinguish between biosecurity and biosafety measures. Biosafety entails the use of containment principles, technologies and practices that are implemented to prevent unintentional exposure to pathogens and toxins, or their accidental release. Whereas biosecurity is intended to deter or detect the loss or theft of dangerous biological materials for illicit or malicious purposes, the biosecurity best practice guidelines focus on preventing unauthorised access to dangerous biological materials in BRCs. They are not intended to address biosecurity in other types of facilities, nor do they address specific measures related to crisis management in the event of a security breach. Although designed for BRCs, the principles of this guidance hold true for anyone holding, handling, utilising and sharing microorganisms.

The microbial domain Biological Resource Centres (mBRCs) have being taking action using the OECD best practice described above as a basis and have developed a code of conduct to help create a safe environment and facilitate research [19] http://ijs.sgmjournals.org/content/63/Pt_7/2374.long. The Microbial Resources Research Infrastructure (MIRRI) on the European Strategy Forum for Research Infrastructures (ESFRI) road map has taken up the challenge to ensure best practice (www.mirri.org). MIRRI is a pan-European distributed research infrastructure that provides facilitated access to high-quality microorganisms, their derivatives, associated data and services for research, development and application. In December 2014, it brought together the collections community with groups representing governments and policy makers, training and education, standards and regulation authorities and the bioscience and bioindustry communities to raise awareness and help put in place practical solutions. MIRRI through its partners is participating in International Standards Organisation (ISO) Technical Committee 276 Biotechnology, designing a set of standards for biotechnology impacting on the provision and use of living materials from biobanks (http://www.iso.org/iso/home/standards_development/list_of_iso_technical_committees/iso_technical_committee.htm?commid=4514241). The additional participation in working group 5 ‘Laboratory biorisk management’ of ISO/TC 212 complements the efforts of MIRRI in the development of essential but yet missing global standards in the field of biobanking. Keeping abreast of such initiatives is essential not only for mBRCs but for the microbiology community in general [9].

At the highest level, e.g. governmental, the goal is to implement measures to protect us against malicious use of microorganisms, their products, information and technology transfer. Terrorists and ill-meaning individuals from inside and outside of the aforementioned organisations could use them in direct or indirect acts against humans, livestock, crops, food, water infrastructure and other economically valuable entities. The threat of someone acquiring a human pathogen for mal-intent is real but to date there is no common agreement of the level of risk, reflecting the likelihood of this happening, and no harmonised system for reporting of adverse incidents. Despite this, such issues impact upon microbiology and its exponents. Keeping abreast of developing issues and the resultant implemented measures that impact upon microbiologist’s daily activities is becoming more difficult. Biotechnology applications continue to grow at a rapid pace, particularly in developing countries. Technical capabilities that were previously concentrated in highly developed countries are increasingly being employed more broadly around the world. In the field of microbial resource collections, the World Federation for Culture Collections (WFCC), who list over 700 culture collections in the World Data Centre for Microorganisms (WDCM—www.wdcm.org) with over 6000 staff around the world helps its members by participating in relevant international initiatives on biosecurity (www.wfcc.info).

Microbiologists need to have a reliable source of authoritative information. Of course the national authority provides the correct in-country information but finding it is not always easy and as regulations and national oversight vary from country to country, the requirements of other countries are not readily available. Although over time, the affected communities have made great strides in understanding and controlling risks occurring from handling microorganisms, these improvements have not been applied consistently or evenly at the regional and global level. The Microbial Resources Research Infrastructure (MIRRI—http://www.mirri.org/home.html) has been working with other stakeholders on this topic and endorses the code of conduct on biosecurity for Biological Resource Centres [19]. MIRRI considers that awareness raising and education are basic requirements to ensure the safe use of microorganisms, more than ever in the absence of standardised and harmonised systems and regulation. A wealth of information is available which can be accessed through several sources; a starting point can be through the OECD-specific website on biosecurity issues (http://www.biosecurity.org). There are several educational tools and courses available for example through the University of Bradford, which has published authoritative books on biological security education addressing undergraduates in life-science courses as well as team-learning-based education handbooks on biological security (see http://www.bradford.ac.uk/social-sciences/peace-studies/research/publications-and-projects/guide-to-biological-security-issues/ ). Rappert and McLeish [17] published the work *A Web of Prevention: Biological Weapons, Life Sciences and the Governance of Research* addressing life-science research and its implications for security. It provides an insight into current discussion on effective preventive measures and effective control measures. There are several web-based tools including new security learning at http://www.newsecuritylearning.com/index.php/archive/78-building-capacity-in-dual-use-bioethics-biosecurity-education-for-life-scientists which provides technology-assisted training for security and defence and emergency services. Other sites are aimed at researchers and practitioners such as those through Bradford University http://www.bradford.ac.uk/research/sustainable-societies/impact/global-biosecurity/.

The following describes the issue of biosecurity, introduces some of the actors and their roles and highlights best practices to reduce the potential for misuse of microorganisms.

### Addressing Biosecurity at the International Level

Eighty years ago, the Geneva Protocol on the prohibition of the use of biological and chemical weapons in war was drawn up. In 1969, the way was paved for the Biological and Toxin Weapons Convention (BTWC). The BTWC forms the baseline agreement that lays down the key principles internationally on disarmament and proliferation control measures. It came into force in its current structure over thirty years ago, on 26 March 1975; 174 countries are States Parties to it. In 2001, following the 9/11 attacks and the Amerithrax the picture changed completely and the term biosecurity evolved, drawing attention to the potential of modern biotechnology to be exploited for malicious ends [5]. A ‘code of conduct’ for scientists (‘professional ethics’) was requested at different levels, e.g. during Australia Group meetings it was regarded necessary to protect the biological weapons control and prevent further possible erosion. It was considered that more effective control mechanisms, data protection and better information on relevant documentation and tracking were required. The Australia Group came into existence in the 1980 s, has grown to include 34 members and encourages countries to impose export measures for control of dual-use goods. This globally important initiative has many outreach activities. It is an informal group of countries committed to combating the proliferation of chemical and biological weapons providing the international community with lists of potential dual-use materials (microorganisms) that need to have controlled access for legitimate use.

The European Union brought in regulations, firstly addressing biosafety through containment dependent on hazard with the aim of reducing health risks associated with microorganisms; the Control of Biological Agents - Health and Safety EC Directive 2000/54/EEC on Biological Agents http://eur-op.eu.int/opnews/395/en/r3633.html. More specifically regarding biosecurity, the EU Council Regulation 3381/94/EEC on the Control of Exports of Dual-Use Goods from the Community http://eur-op.eu.int/opnews/395/en/r3633.html was introduced to control access to organisms that could be misused. At the time, the culture collection community in Europe initiated activities to raise awareness of these issues and improve practices to address the regulations and the need to control access to legitimate uses. In particular, the European Commission funded project European Biological Resource Centres Network (EBRCN) issued information resource documents now available via the WFCC website www.wfcc.info.

The Inter-Academy Panel on International Issues (IAP), the International Council for Science (ICSU) and The National Academies of the United States *International Forum on Biosecurity* met 20–22 March 2005 in Como, Italy. The discussion reflected concern over the growing awareness that rapid developments in the life sciences and biomedical research, while offering great benefits, also pose the risk that the knowledge, tools and techniques that enable these advances might be misused to cause deliberate harm. Any effort to address this ‘dual-use’ dilemma must ultimately be international, since biotechnology research is a genuinely global enterprise. The scientific community has an essential role in ensuring that efforts to manage the risks do so in a way that fosters both improved security and strengthened international collaboration to ensure scientific advances.

The Chemical and Biological Arms Control Institute (CBACI) and the International Institute for Strategic Studies—USA (IISS-USA) conducted a joint project to promote the engagement of the global biotechnology industry in issues of public safety and security with special attention to biological weapons and bioterrorism. The CBACI is a policy research organisation established in 1993 to address the challenges to global security and stability with a special, but not exclusive focus on the elimination of chemical weapons and biological weapon. The IISS promotes the development of sound policies that further global peace and security and maintain ‘civilised’ international relations. The project has resulted in the creation of the International Council for the Life Sciences (http://www.embo.org/scisoc/icls_charter.pdf), a global organisation of biotechnology and pharmaceutical firms and other entities to establish a self-sustaining enterprise that provides a mechanism for private industry to contribute to improved quality of life and to enhance international standards of public safety and security on a global scale through responsible, ethical and sound business and scientific practices and to facilitate the development of effective partnerships between the life-sciences industry, government, international organisations, the scientific community and other critical constituencies on these vital issues of common concern. Their discussions have raised some important views:

- Biological, Chemical and Nuclear dangers are huge.
- Terror groups can process biological agents for misuse.
- Governments cannot address issues alone and need international collaboration.
- The outcome of the event of an attack will depend on preparedness.
- Impacts of biological, chemical and nuclear dangers extend beyond humans and include animals, plants and the environment.
- All microbiologists must follow best practice to keep pathogens out of the hands of those who may misuse them.
- Scientists must not sit back and wait for an event that will stimulate reaction, and there is a need to be proactive in prevention and preparedness.
- Health security will depend upon public/private cooperation.

A key issue is the capability and intent for bioterrorism. There might be a shortening in timelines between the idea, intent and potential to actual capacity and ability to carry out a bioterrorist act, fuelled by the availability of tools, technology and information. A charter to be adopted by the biotechnology companies, organisations and other producers and suppliers has been published on the International Council for the Life Sciences (ICLS) website, and interested parties are invited to participate (http://www.iclscharter.org/).

While in most high consequence industries (e.g. nuclear industry, airline industry, extracting and chemical industries) proactive risk management has been widely implemented, there is a need for a universally accepted system for biorisk management in life sciences including good mechanisms to provide appropriate risk communication. The microbiologist community should consider adopting the self-conception of their scientific work as being part of such a high consequence community. This basic understanding is living awareness and being aware that the bioterrorism threat is something that microbiologists must address and effectively deal with alongside a number of similar issues that require similar controls, e.g. compliance with national legislation and international conventions, handling of emerging diseases, health and safety, access to genetic resources and security. Bioterrorism can impact on the way microbiology is undertaken but it mustn’t impede its progress. It is therefore essential that measures to reduce such impact are embedded in normal operational practice as far as is possible. Biorisk assessment needs to be carried out for several reasons for health and safety, for transport regulation and to identify dual-use organisms, the assessment should indicate what needs to be implemented for each microorganism handled. The OECD guidance for BRC attempts to do this but relies upon resources such as the World Health Organisation’s Laboratory Biosafety Manual third edition [23]. This provides a key source for biosafety support and information to practitioners in microbiology. The WHO has also produced biorisk management, laboratory biosecurity guidance [24] which helps BRCs understand their responsibilities in risk assessment. Above this operational level best practice scientific societies and organisations such as the International Union of Microbiological Societies [8] and the European Culture Collections’ Organisation [18] have endorsed codes of ethics or conduct to raise awareness and to introduce safe practices.

The OECD Biological Resource Centre (BRC) initiative drafted guidance to deliver a practical approach that enables legitimate research and development but reduces the opportunity for misuse. The OECD Biological Resource Centre (BRC) Biosecurity Guidance was published in 2007 [15] which left some questions still to be answered around biorisk assessment. There are a defined number of human pathogens but even here an agreed international list of organisms is difficult to achieve. The organisms of biosecurity concern extend beyond the human pathogens to include crop and animal pathogens and those that can be used to threaten environmental and economic targets. The OECD BRC Task Force agreed that guidance was necessary but that it should not be bureaucratic and applied to situations that don’t require it. The basic principles of the guidance are that BRCs should:

- Be accredited/certified to handle organisms to a specific hazard level.
- Comply with legislation over national boundaries.
- Not increase the hazard level of the organisms they hold.
- Enable full traceability of distribution—i.e. the requirement for MTAs material transfer agreements and end-user certificates.

It would therefore follow that only approved BRCs could hold agents of concern and that exchanges across national boundaries would be between BRCs of equal clearance. The OECD provides information that extends beyond the BRC community and has created a web-based information resource (http://www.biosecurity.org).

There is an argument that the threat is mostly economic, the target may not be human. In the light of what happened in Asia with SARS and bird-flu, the consequence of a disease outbreak (human, animal or plant disease), whether occurring naturally or with intended or involuntary human intervention, is serious injury, economic loss, productivity loss and even death. As national controls are put in place through national legislation, for example the Patriot Act in the USA and Counter-Terrorism and Security Act 2015 in the UK, the lists of organisms of concern differ. The effect of human pathogens is global, but this is not the case for crop pathogens and what might be a threat in one country will not be in another. In a spectrum of risk, spanning natural events, from emerging disease through man’s intervention, e.g. from laboratory accidents to deliberate acts, to bioterrorism, the greatest risk comes from emerging disease. There are several groups working on risk assessment, and publications are available on these issues, e.g. Hood et al [6] The Government of Risk: Understanding Risk Regulation Regimes. It was considered by participants of the MIRRI workshop ‘biosecurity implementation strategies and compliance management in mBRCs’, 1–3 December 2014, that the number of organisms is small and the likelihood of an event rare but a biorisk assessment carried out should indicate the organisms of concern, in which circumstances and consequently the precautions that need to be taken. The authors believe that such requirements be built into normal operations of the laboratory and become routine. MIRRI assists in introducing an environment of compliance and helping its partners by developing best practices.

In the light of increasing control of access to microorganisms and their safe handling by national and international laws, regulation, best practice and international standards, microbiologists need to be able to select the most appropriate tools for their specific use. Facilitating compliance and a safe system for access and distribution of microorganisms does not restrict legitimate use and shall always follow a principle of appropriateness, which does not demand the same rules for materials that present little risk.

### Role of Training and Education in Raising Awareness of the Need for Legal Compliance and Implementing Best Practice

The life-science community is a key stakeholder in the effort to ensure that the advances in biotechnology are not misused for hostile purposes. Unfortunately, to date, the engagement of life scientists with issues of biosecurity has been limited [1]. The ongoing debate on the risks and benefits of gain-of-function research is a notable exception in this regard. Generally, studies and surveys carried out over the past decade have demonstrated that the level of awareness of biosecurity among life scientists is low. This is hardly surprising, since biosecurity issues seldom feature in the formal life-science curricula [10].

Measures for promoting biosecurity awareness in the life sciences have been the subject of discussion within the framework of the BTWC for a number of years now. Codes of conduct and training programmes were among the topics under consideration among States Parties to the Convention during the 2007–2010 Inter-Sessional Process [2]. The Seventh Review Conference of the BTWC held in December 2011 agreed on the value of national implementation measures to:

1. implement voluntary management standards on biosafety and biosecurity;
2. encourage the consideration of development of appropriate arrangements to promote awareness among relevant professionals in the private and public sectors and throughout relevant scientific and administrative activities;
3. promote among those working in the biological sciences awareness of the obligations of States Parties under the Convention, as well as relevant national legislation and guidelines;
4. promote the development of training and education programmes for those granted access to biological agents and toxins relevant to the Convention and for those with the knowledge or capacity to modify such agents and toxins;
5. encourage the promotion of a culture of responsibility among relevant national professionals and the voluntary development, adoption and promulgation of codes of conduct [3].

Relevant projects, initiatives and proposals have been presented and put forward both during the formal deliberations of States Parties and during side events and panel discussions held on the margins of the BTWC Meetings [16].

The need for fostering biosecurity awareness among the life-science community has further been underscored in numerous authoritative high-level reports, which have identified it as one of their key recommendations. Some notable examples include:

- *Biotechnology Research in an Age of Terrorism* (Fink Committee Report), US National Research Council, 2004 [12];
- *Globalisation, Biosecurity and the Future of Life Sciences* (Lemon-Relman Committee Report), US National Research Council, 2006 [13];
- *Brainwaves Module 3: Neuroscience, Conflict and Security*, UK Royal Society, 2012 [21];
- *Improving Biosecurity: Assessment of Dual*-*Use Research,* Royal Netherlands Academy of Arts and Sciences, 2013 [20];
- *Biosecurity*—*Freedom and Responsibility of Research,* German Ethics Council, 2014 [4].

Some progress has already been made in the area of content development for biosecurity education and training programmes. The work carried out by the Biosecurity Working Group of the Inter-Academy Panel (IAP)—The Global Network of Science Academies, US National Academies of Sciences (US NAS) and University of Bradford, UK, is indicative in this regard. In 2005, the IAP published a Statement on Biosecurity which outlined a set of fundamental principles that could serve as a basis for the development of codes of conduct [7]. At the time when the Statement was issued, codes of conduct were among the key tools considered as a means of raising awareness of the BTWC and the broader social, legal and ethical implications of novel life-science advances. The US NAS have completed a number of projects focusing on building capacity in the area of responsible science. Through the implementation of institutes across the Middle East and North Africa (MENA) and the South-East Asia regions, US NAS have successfully empowered faculty members specialising in the life sciences with knowledge and skills that can help embed responsible science education at a local level [14]. For its part, the University of Bradford has worked on a number of projects aimed at developing biosecurity training content, with the most recent initiative encompassing the production of a twofold online educational resource (www.bradford.ac.uk/research/sustainable-societies/impact/global-biosecurity/). The resource comprises a Guide to Biological Security Issues titled *Preventing Biological Threats: What You Can Do* [22]. Its twenty-one chapters provide a detailed overview of the security challenges arising from the rapid progress of biotechnology; the international biological prohibition regime aimed to ensure that the life sciences are utilised only for peaceful, prophylactic and protective purposes; the role that different stakeholders, such as scientific organisations, industry, the law enforcement community and governments can play in the implementation of biosecurity. The Guide further highlights the significance of applying active learning methods when teaching biosecurity. The book builds on the wealth of experience of the authors and is intended to raise awareness and knowledge of biological security of everyone active in the life sciences including those engaged in research to those engaged in management and policy-making at both the national and international levels. In order to facilitate the dissemination of training content, the Guide is accompanied by a manual, *Biological Security Education Handbook: The Power of Team*-*Based Learning* [11]. The Handbook seeks to assist lecturers and trainers with the development of biosecurity courses and seminars using a cutting-edge active learning approach—Team-Based Learning. The format has been specifically selected, not least because of its user-friendly structure and proven efficiency and effectiveness for various purposes in different educational settings. Both books are freely available online and are currently being translated in Arabic, Russian and Ukrainian.

A crucial factor that is likely to have far-reaching implications for the demand of biosecurity education and training is the growing attention to the articulation and introduction of professional competence standards for life-science practitioners. In 2015 the International Federation of Biosafety Associations (IFBA) launched an international certification programme which is intended to fulfil IFBA’s ‘mission of safe, secure and responsible work with biological materials’ (http://www.internationalbiosafety.org/index.php/news-events/news-events/news-items/470-ifba-launches-certification-program-for-biorisk-management-professionals). Among the relevant qualifications that life-science professionals can take, one is exclusively focused on biosecurity, covering a broad spectre of issues related to international regulations and guidelines; risk assessment; personnel reliability; physical biosecurity measures; pathogen accountability; and dual use and bioethics (http://www.internationalbiosafety.org/index.php/professional-certification/professional-certification/studying).

While biosecurity awareness is certainly an essential condition for minimising the risks of the hostile misuse of the life sciences, it is important to note that it is neither a sufficient measure, nor a ‘silver bullet’. Rather, it needs to be considered and promoted as part of a broader complex of relevant policies and mechanisms, designed to foster a robust biosecurity culture, and thus sustain an integrated and comprehensive web of preventive measures which discourages the misuse of microorganisms for hostile purposes.

### MIRRI’s Approach to Compliance and the Introduction of Safe Practices

To develop safe practices beyond awareness raising MIRRI has adopted the OECD best Practices on Biosecurity for BRCs (OECD 2007) and the code of conduct for mBRCs [19]. These will be implemented through its Partner Charter when the legal entity for MIRRI is established. A Policy statement on Biorisk assessment in Biological Resource Centres and implementation of biosecurity measures was submitted to the European Commission as part of its preparatory phase European Union’s Seventh Framework Programme project output. This was based on the analysis of the results obtained from a biosecurity questionnaire and risk assessment trials carried out with partners spanning 11 European Union countries. In addition, MIRRI organised a workshop on biosecurity implementation strategies and compliance management in mBRCs, 1–3 December 2014. Participants represented industry, academia, experts in biosecurity, policy makers and microbiologists including those from mBRCs. There were a number of issues identified; to resolve these it was recommended that a general task force focussing on the implementation and impact of all relevant guidance documents should be set up. In addition a consensus view on how these might be implemented is required. There were several other issues and concerns raised through the biosecurity survey questionnaire that need resolution. Education and training were considered to play a crucial role in the implementation of biosecurity demands; this should include curricula modules for academia/ universities as well as being an element in continued professional development. Establishing defined programmes for this was desirable while taking advantage of the existing programmes such as the Bradford training programme which provides a very intensively elaborated approach to the topic. It was clear that all participants supported increased communication between institutions and between mBRCs in particular. Participants learned that the Dutch Government had taken the lead in providing its scientists with support in biosecurity. An office dedicated to biosecurity issues had been established in the Netherlands (http://www.bureaubiosecurity.nl/en). It is a temporary national information centre that provides a toolkit to help determine the level of biosecurity that is needed and provides information on best practice. This was viewed as an exemplary and commendable model that should be built upon across Europe. Laboratory biorisk assessment and the active processes were considered a difficult facet of biosecurity in daily practice; help is still needed in this aspect.

Providing quality to the recipients of bioresources was raised to be fundamentally important; this requires proper risk assessment and useful standards and regulatory guidance. Access to highly pathogenic bacteria allocated to the Risk Group 3 was getting more difficult to acquire and not maintained by many mBRCs. In order to carry out the necessary research on such disease-causing organisms, it was apparent that the causative organisms will need more attention in the future. It remains a matter of importance that research must not be restricted, and it would be counter-productive. Dual-Use-Research-of-Concern has issues over scientific research that is intended to be utilised for a beneficial purpose but that can provide knowledge, information, products or technologies that could be directly misapplied to pose a significant threat to public health and safety, agricultural crops and other plants, animals, the environment, materiel or national security. Participants discussed the balance between publishing science and trying to reduce the potential of its uptake and misuse. Consensus remains that the benefits of publication of such research outweigh the risks, and that suppression of information impedes science and development. The most recent development on establishing a new ISO Standard (ISO/TC 212) on the basis of CWA 15793 on Laboratory biorisk management for laboratories highlighted that a broad dialogue, alliances and transparency seems highly relevant. MIRRI has the unique chance and should play an important role in solving the demand for standardised risk assessment, legal and governmental support, alignment of interests and generation of coherence and harmonisation. Thus, MIRRI developed a road map to address these demands and intends to establish an expert cluster, as well as contacts to governments to promote the establishment of biosecurity offices.

The key elements of the subsequent established MIRRI policy on Biorisk Management in mBRCs are:

1. Follow the relevant national law and adhere to,
  - the code of conduct on biosecurity for BRCs,
  - other comparable recognised standards,
  - OECD Best Practice Guidelines on biosecurity for BRCs.
2. Follow the development of biosecurity implementation strategies and adjust practice accordingly.
3. Work in collaboration with MIRRI and external partners towards developing and implementing protocols for adequate biosecurity risk assessment of holdings and normative compliance in MIRRI-mBRCs.
4. Offer available specific expertise to the MIRRI biosecurity expert cluster.
5. Work with national authorities to increase competence and advocate the establishment of national biosecurity offices and their international cooperation.
6. Work in collaboration with MIRRI and external partners to strengthen the ethical basis for biosecurity in the scientific community.
7. Adopt existing or develop new educational tools to raise awareness among mBRC staff.

The MIRRI strategy for the implementation of biosecurity measures is based on the determination of risk levels (profiles) as a result of risk assessment and the establishment of an institutional biorisk policy with relevance to risk prevention. These elements lead to measures in biosecurity, which need to be implemented via harmonised procedures and monitored within a continuous improvement process. Comprehensive biorisk management covers both complementary elements, risk assessment and risk prevention and governs the required procedures using standard management system tools. MIRRI encourages the appointment of a responsible biosecurity officer or biorisk professional to be in charge of the integration and improvement of biorisk management and the risk communication with staff and third parties.

**Fig. 1:**
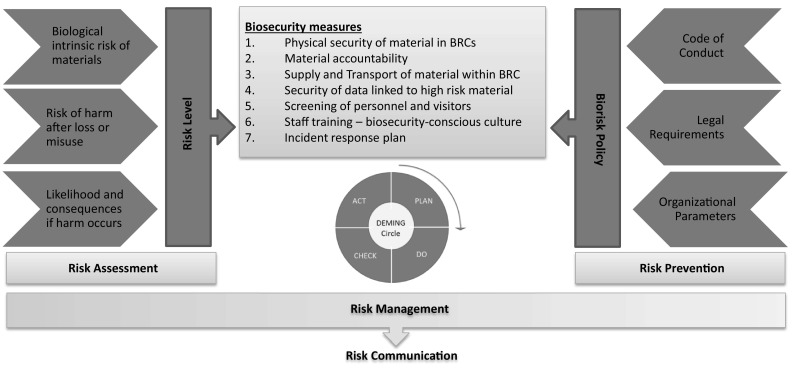
MIRRI biorisk management system

Following this scheme, the identified main biosecurity measures (Fig. 1) are:

1. Physical security of material.
2. Material accountability.
3. Supply and transport of material.
4. Security of data linked to high risk material.
5. Screening of personnel and visitors.
6. Staff training—biosecurity-conscious culture.
7. Incident response plan.

The MIRRI biorisk management system is based on a management system approach, which enables an mBRC and other organisations handling biological material to effectively identify, assess and control the biosafety, biocontainment and biosecurity risks inherent in its activities. The biorisk management system is built on the concept of continual improvement through a cycle of planning, implementing, reviewing and improving the processes, measures and policies that an organisation establishes to meet its goals. The systematic evaluation and correction of this system leads to improved performance and control of biorisks.

## Conclusions

Biosecurity is a shared responsibility of government, science, industry and the community who must all work together to implement best practices without impeding our ability to undertake science. Bringing together the stakeholders of biosecurity issues and establishing a unifying biosecurity culture as well as compliance understanding, lays the foundation for the implementation of strategies and best practices to minimise the risks and dangers that could arise from any use of pathogenic biological material in any stage of handling during science or commercial research and development.

Discussions on the most appropriate risk management procedures that are feasible in the daily work of a microbiologist continue. The organisational background and managerial capacities of the institutions carrying out such work must be considered in the choice of best practice to ensure they are not beyond abilities to implement them. The governance of risks is a question of organisational development embedded in a supportive socio-economic environment; risk management must therefore follow reasonable principles and realistic implementation steps adjusted to the individual situation. A well-considered approach has the potential to overcome the reserved attitude of many microbiologists towards engagement in biorisk management. Ultimately, the goal is a realisable but nevertheless effective system where one does what one can with the means available.

The authors strongly believe that scientists wish to comply with regulatory requirements but primarily wish to focus on their work in search of discovery. They need to be supported in this at the institutional, community, national and international level with clear and practical best practice. Where possible this needs to be built into routine of the day to day work as much as possible. They need to be made aware of the reasons for the implementation of these best practices. Fundamentally, such practices must be in-built at the beginning of careers and through educational processes. Educational and vocational training plays an important role in processes leading to safe and ethical science.

During its implementation phase MIRRI will define best practices for the different risk levels, set up harmonised procedures and establish a network of experts across Europe supporting mBRCs in their individual implementation of a biorisk management. The envisaged virtual working platform MIRRI Coordinated Work Environment (CWE) will be a beneficial tool for knowledge and information exchange. Best practice to ensure biosecurity will be a requirement of mBRCs under the MIRRI partner charter, and the envisaged expert cluster will provide advice and where possible, solutions to the problems raised by mBRCs and microbiologists in general, particularly those raised during the biosecurity survey. In the meantime microbiologists in general should make themselves aware of their responsibility in the prevention of malpractice in the use and application of microorganisms by utilising the educational tools available, observing the code of conduct on biosecurity for Biological Resource Centres and implementing best practice in their work.
